# Liquid Crystal Microlenses for Autostereoscopic Displays

**DOI:** 10.3390/ma9010036

**Published:** 2016-01-11

**Authors:** José Francisco Algorri, Virginia Urruchi, Braulio García-Cámara, José M. Sánchez-Pena

**Affiliations:** Electronic Technology Department, Carlos III University of Madrid, Avenida de la Universidad 30, Leganés E28911, Spain; vurruchi@ing.uc3m.es (V.U.); brgarcia@ing.uc3m.es (B.G.-C.); jmpena@ing.uc3m.es (J.M.S.-P.)

**Keywords:** autostereoscopic display, spatial multiplexing, liquid crystal microlenses

## Abstract

Three-dimensional vision has acquired great importance in the audiovisual industry in the past ten years. Despite this, the first generation of autostereoscopic displays failed to generate enough consumer excitement. Some reasons are little 3D content and performance issues. For this reason, an exponential increase in three-dimensional vision research has occurred in the last few years. In this review, a study of the historical impact of the most important technologies has been performed. This study is carried out in terms of research manuscripts per year. The results reveal that research on spatial multiplexing technique is increasing considerably and today is the most studied. For this reason, the state of the art of this technique is presented. The use of microlenses seems to be the most successful method to obtain autostereoscopic vision. When they are fabricated with liquid crystal materials, extended capabilities are produced. Among the numerous techniques for manufacturing liquid crystal microlenses, this review covers the most viable designs for its use in autostereoscopic displays. For this reason, some of the most important topologies and their relation with autostereoscopic displays are presented. Finally, the challenges in some recent applications, such as portable devices, and the future of three-dimensional displays based on liquid crystal microlenses are outlined.

## 1. Introduction

**Figure 1:**
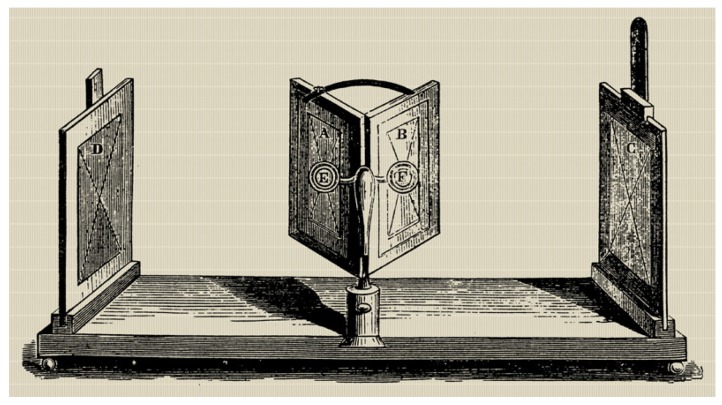
Charles Wheatstone-mirror stereoscope (XIX century) [1]. Public Domain.

More than 170 years ago, Charles Wheatstone demonstrated the first stereoscope to the Royal Society (1838) [1]. He illustrated the basic principle of stereoscopic vision, the brain’s capacity to achieve depth perception from different images. He combined mirrors to present separate images representing different perspectives of the same object for each eye (Figure 1). A few years later, Rollman proposed the use of anaglyphs in order to display stereoscopic pairs [2]. The barrier technique was later proposed by both Jacobson and Berthier around 1896 and it was first applied by Ives in 1903 [3]. He called it the “Parallax Stereogram“. Later, in 1908, a new technique based on using spherical lenses instead of opaque barrier lines were proposed by Lippmann [4]. This was called “La Photographie Integral”. Lippman was a man before his time and this invention could not be further developed because of the technical limitations at the time. Some researchers, such as Herbert Ives, simplified this technique in 1930 by using a lenticular lens array [5]. These images were of poor quality due to the production methods available at the time. In the 1950s, three-dimensional (3D) vision was attempted to be displayed in some cinemas by using anaglyphs. The low quality of the content led to a decrease in interest. In the 1970s, the system was improved thanks to the patented system of Stephen Gibson “Deep Vision” [6]; red-cyan anaglyphs could reproduce the color of the skin better than the red-blue or red-green anaglyphs. Despite this, it was not until the 1980s when better results were achieved thanks to IMAX (Image Maximum) high-resolution displays and the use of linearly polarized glasses. The introduction of these systems produced a great commercial impact that increased the interest of research groups in this field.

**Figure 2:**
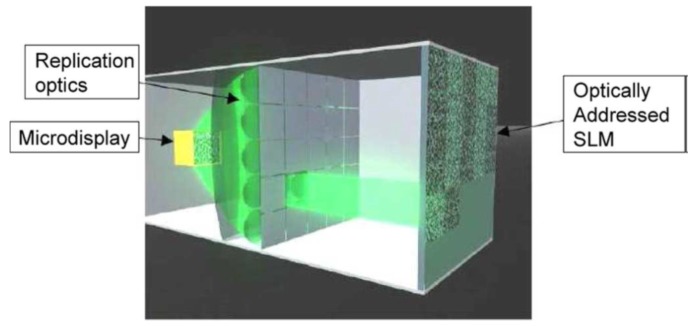
One channel of the active tiling modulator concept of QinetiQ [8]. Reproduced with permission from Slinger, C.; Cameron, C.; Stanley, M. Computer; published by IEEE, 2005.

Currently, 3D technology can be classified into two major groups: one requiring the help of external means, normally glasses (stereoscopic), and another that allows the generation of 3D vision without their need (autostereoscopic). As can be deduced, the use of autostereoscopic devices is the current objective of research groups. These systems were investigated for the first time by Ives (1903) and Lippman (1908), using a barrier system and microlens, respectively. However, technological limitations stopped their development. More recently, this technology has improved considerably, primarily due to new manufacturing processes, such as advanced photolithography techniques, and the reduction of production costs. Furthermore, this technology can now display 3D and different styles of animated visual effects without the requirement of special viewing glasses. Among the multiple techniques on autostereoscopic displays, there are three major lines of research:

*Electro-holographic* is a diffraction-based coherent imaging technique [7]. These systems are based on the phase difference produced when different optical wavelengths are reflected by a certain object. In this type of system, a specific object can be reproduced from a flat 2D display that possess complex transparency representing amplitude and phase values (Figure 2). This technique is one of the most promising because it can reproduce 3D vision with full parallax and without the problems of convergence and accommodation. This is also the most challenging technique because it requires coherent sources and very high resolution spatial light modulators. Interest in this technology has increased exponentially in the last years.

**Figure 3:**
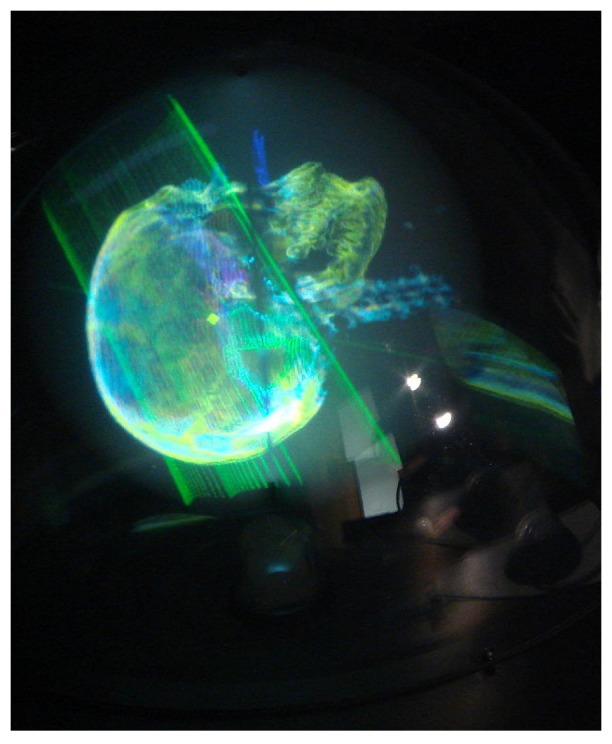
“PerspectaRAD mouse Phantom” by Gregg Favalora. Licensed under CC BY-SA 3.0 via Commons.

*Volumetric* technique creates the 3D image by projecting the rays onto a volume (Figure 3) or using discrete locations of luminescence within a volume [9]. There is no officially accepted topology because of the variety of volumetric displays. They can be based on the eye persistence (swept-volume display) or using laser light to encourage visible radiation in a gas (static volume). The main problems are the use of movable parts and the low resolution. Moreover, a large amount of data is necessary to store the 3D image.

**Figure 4:**
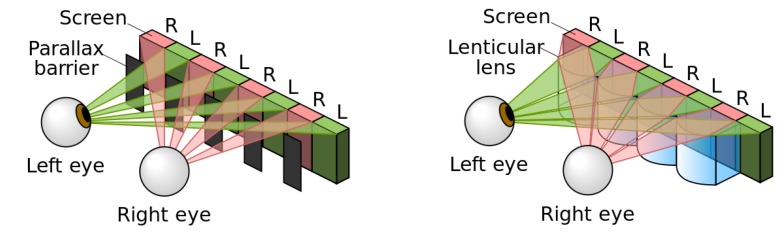
“Parallax barrier *vs.* lenticular screen” by Cmg Lee. Licensed under CC BY-SA 3.0 via Commons.

*Spatial multiplexing* is based on multiplexed 2D images. There are two main techniques: time and spatial multiplexing. In the first case, the 2D image reach one eye at different times, exploiting the persistence of the human visual system to give 3D perception. This technique usually needs spatial light modulators and active shutters [10] or special backlights [11] or simply dynamic displays [12]. The main advantage is the full resolution of the displayed images, while some common problems are moiré patterns [13], crosstalk [14] and flicker [15]. For this reason, this technique is in the research stage and no commercial displays have been produced. Spatial multiplexing techniques apply optical phenomena including diffraction, reflection, refraction and occlusion in order to deviate the images to certain positions in front of the display. Among the different technologies to achieve this effect, refractive elements (microlenses) and occlusive elements (parallax barrier) are the most successful. A parallax barrier consists of masks that contain vertical apertures to cover the light at certain angles (see Figure 4, left). The barrier can be considered a mature technology because commercial devices have already been produced. For example, dynamic barriers based on Liquid Crystal Displays (LCDs) are used to generate black and white columns. Despite its maturity, this technology has considerable drawbacks such as lower brightness, small viewing angles, and crosstalk, which is caused by diffraction and it is difficult to suppress [16]. To improve the function of this type of system, lenticular technology can be used (see Figure 4, right). In this case, microlenses deviate the light to certain directions corresponding to each different eye.

**Figure 5:**
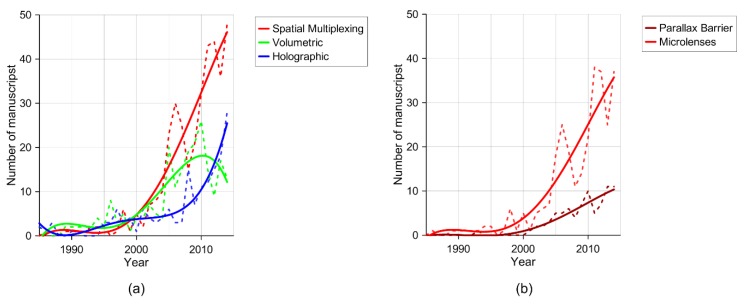
Number of manuscripts as a result of the search in Scopus database (Elsevier). The search was restricted to only the titles of the manuscripts: (**a**) Autostereoscopic technologies and (**b**) Spatial multiplexing technologies. Queries for each technology were “holographic and display and 3D (or three-dimensional or autostereoscopic)”, and “volumetric display” and for spatial multiplexing, “parallax barrier display” and “lenticular (or lenses or microlenses) and display”. Dashed lines are real data, while solid lines are tendency lines (polynomial fit). The fitting is done by an orthogonal polynomial with four degrees.

In this review, a study of the impact of these three technologies over the last few years has been carried out. In Figure 5a, the three main lines of research mentioned above are classified in terms of research manuscripts per year (source: Scopus). Interesting results arise from this survey. Among the three techniques, spatial multiplexing is the one with the largest scientific production. In fact, this technique is the most successful in terms of efficiency and quality. Most of the commercial autostereoscopic displays are based on this technique and some of the most promising research is focused on this line (integral imaging). In Figure 5b, the works devoted to the two main solutions to implement this technology (parallax barriers and microlenses) are studied independently. Due to the disadvantages of parallax barriers mentioned above, the research of this technology has been developed to a lesser extent in comparison to lenticular devices based on microlenses. Holographic technique is still in the research stage. Despite this, as can be seen in Figure 5a, the number of scientific manuscripts in this field has greatly increased in recent years. This is probably the most realistic 3D reproduction but the technical problems are still numerous. The tendency indicates that in two or three years the amount of research will be similar to spatial multiplexing techniques. Finally, volumetric research peaked few years ago, but the survey seems to suggest a decreasing interest in this technique.

As stated above, the most researched technique is actually spatial multiplexing. Moreover, Liquid Crystal (LC) lenses may be used to improve the characteristics of this technique. In this review, first we discuss the technical issues of spatial multiplexing techniques used actually. Some commercial products and their technical characteristics are presented in Section 2. The use of microlenses is clearly stated. For this reason, in Section 3 a complete study of LC microlenses, focused on their use in autostereoscopic devices, is presented. Finally, a critical analysis is included in Section 4.

## 2. State of the Art of Spatial Multiplexing Technique

This technique is currently used in several commercial products and seems to be the most promising for giving rise to future products in the market. The basic scheme is based on the optimization of two aspects: the capture of a stereoscopic pair of images stereogram for display on a screen and the correct visualization of these images by the user. This configuration is known as a two-view autostereoscopic display. The stereograms consist of images (corresponding to the same scene viewed from two different angles) that are intertwined in columns of pixels. At this point, a system capable of directing the path of light of each column of pixels, to the corresponding eye, is necessary. Then, the brain interprets the signals and generates a 3D virtual image. However, there are some practical problems. The viewer must stay in the right position or a pseudoscopic image is produced. Moreover, eyestrain may occur caused by a mismatch between convergence and accommodation. This is an unnatural condition that disrupts the correlation between vergence and focal distance [17]. The larger the depth is, the larger the visual strain becomes. These limitations can be overpassed by increasing the number of views and using full parallax (multi-view [18] or integral imaging [19]). In multiview displays, multiple different images are presented in front of the display. Still, the number of views is too small for continuous motion parallax. Some proposals have been made to minimize the transitions between views [20]. Still, no full parallax is provided. Integral imaging has both characteristics, motion and full parallax. Nevertheless, such technique proposed by Lippman in 1908 have been recently possible from a technical point of view. The need of ultra-high resolution displays, fast processors for processing the large amount of data and a poor quality of the microlens arrays are some reasons of this delay. Integral imaging is the most promising of all spatial multiplexing techniques as it achieves to reproduce a 3D field view similar as it is reproduced in nature [19].

The two main approaches to achieve spatial multiplexing: parallax barriers [21] and lenticular systems [22]. The barriers can be considered a mature technology because some commercial devices based on them have been produced. For instance, dynamic parallax barriers were proposed in order to change the properties of the virtual image (3D distance, number of views, *etc.*) [23]. This technology has considerable drawbacks that can be overpassed by using lenticular lenses.

As mentioned above, parallax barriers and fixed lenticular systems (no based on LC), can already be found in commercial products. This year, (6–9 January 2015), Toshiba presented at the “2015 International Consumer Electronics Show” in Las Vegas a new display using LC lenses. Toshiba plans to fuse the new technology with a partial 2D/3D switching function which can be applied to any screen size and position, aiming for its rapid commercialization in industrial and medical products that require glasses-free high-definition 3D displays. Despite this announcement, the information of the technology behind is still unknown. This is one of the first commercial displays using LC lenses. Table 1 also shows two representative examples of 3D displays already on the market, developed by Sharp and Toshiba.

**Table 1:** Commercial examples of autostereoscopic devices based on spatial multiplexing.

Product	Company	Technology	Resolution	Mode	Year
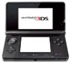	Sharp 3.4″ (3DS)	Dynamic parallax	480 × 854	2D/3D	2011
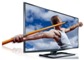	Toshiba 55″ (55ZL2G)	Fixed Lenticular array	3840 × 2160 (4K)	2D/3D	2012
Unknown	Toshiba 15″ (Unknown)	LC Lenticular array	4K	2D/3D/4K	2015–2016

The device of Sharp uses a dynamic parallax barrier that only a few years ago was in the research stage. It is based on the use of LCs and it has many advantages, such as 2D/3D switching, multiple views, variable focal length, wide viewing zone, *etc.* The Toshiba display is based on a set of microlenses of high quality, probably manufactured with some kind of resin or polymer. This display uses a specific eight-core processor that allows calculations of the required views as a function of the number of observers. Some common disadvantages of spatial multiplexing systems have been solved in different ways:

- The loss of resolution is solved through very high-resolution displays (QFHD (quad full high definition), 3840 × 2160 pixels). Moreover, the lenses are placed with a small angle to distribute the loss of resolution vertically and horizontally [24].
- Another common problem is the non-uniformity of the light coming from pixels. The problem is caused by the darker spaces of the pixels that generate bright and dark areas. An observer looking at different angles will notice a change in the image brightness under certain positions. There are three methods to solve this: place the lenses with a convenient angle, adapt the focal distance, and modify the width of the lenses to obtain fractionated views [25].
- Another significant problem are aberrations caused by manufacturing defects. Aberrations cause a non-uniform distribution of the intensity. However, thanks to improvements in various processes, the non-uniformity of the lenses can reduce the crosstalk to values ranging from 2%–7% [26].

Previous systems are patented or commercially available. However, there are still many problems to be solved. Thus, autostereoscopic vision has become a topical issue that requires numerous research resources. In the field of lenticular arrays, the possibility of using LC lenses is being actively investigated. Within LC, lenses can be separated into active (birefringent active) and passive (activated by polarization). Table 2 summarizes a comparison between technologies. Brightness 2D and 3D indicate the total brightness of the display when 2D or 3D images are reproduced, respectively. In autostereoscopic vision, the crosstalk parameter is used to measure the quality of the generated stereoscopic image [27]. Voltage 2D and 3D are the necessary applied voltage to maintain a 2D and a 3D image, respectively. Collected data are the best characteristics found in the bibliography for each of them. It is likely that one of these topologies is part of the new Toshiba display. It must be borne in mind that fixed microlens technologies and fixed barriers can be found implemented in commercial products, while devices based on LC are still part of the research field. Given the great commercial impact of these technologies, they will probably be marketed soon.

**Table 2:** Comparison between different autostereoscopic technologies.

Characteristic	Fixed Parallax	Dynamic Parallax	Passive Parallax	Fixed Lenses	Active Lenses	Passive Lenses
Brightness 2D *	45%	45%–85%	45%	>95%	>95%	>95%
Brightness 3D *	45%	<20%	<20%	>95%	>95%	>95%
Contrast	1:1000	-	-	1:1500	-	-
Crosstalk	>2%–3%	>2%–3%	>2%–3%	<1%	<1%	<1%
Thickness	0.5 mm	1.13 mm	1.65 mm	0.7 mm	1 mm	1.1 mm
Voltage 2D	-	0 V	0 V	-	Depend on the topology	0 V
Voltage 3D	-	3.3 V	3.3 V	-	0V	3.3 V
Switching time	-	<100 ms	<100 ms	-	Several seconds	<100 ms
Multiplexing of 2D/3D areas	-	Differences ×3–×5 in brightness	Differences ×2 in brightness	-	Yes	Yes

* Over a maximum brightness of 500 cd/m^2^.

In this study, different existing technologies for autostereoscopic applications have been explored. It has been found that the only system that allows a tuning of the 3D distance is the method of dynamic parallax barrier with the disadvantages that this system entails. Devices that employ LC lenses are only designed for 2D/3D operation and usually are based on curved surface (explained in next section). There are numerous techniques for manufacturing LC lenses that could be of clear advantage to use in autostereoscopic displays. For instance, patterned electrode is the most promising technique for small pixel sizes. The main problem caused by the low speed of LC lenses could be solved by using stacks of lenses [28]. Aberrations that are a common drawback in these devices could be compensated for by using an aberration compensator devices for rectangular apertures [29]. Besides, the temperature dependence of the LC birefringence can be controlled by special temperature sensors for this type of structures [30,31]. In some cases, when the pixel size is bigger than certain diameter (>200 µm) other topologies would be necessary (curved surface or modal control) [32].

## 3. Liquid Crystal Microlenses for Autostereoscopic Displays

A homogeneous conventional lens has two physical characteristics that contribute to the way in which such lenses modify a wavefront passing through it: the difference between the refractive index of the lens material and the surrounding environment, and the curvature of their interfaces. In addition, it is known that when light travels through a non-homogeneous medium, the wavefronts decrease their speed in the optically dense regions and accelerate in areas of lower density. Therefore, it should be possible to design a lens without curvature but with a material that has a gradient in the refractive index; this device is known as a GRIN (Gradient Index) lens. This is the main operating principle of LC lenses. Liquid crystals are optical anisotropic materials with birefringence that can be controlled by an applied voltage. Thus, most of the proposed LC lenses have a graded refractive index produced by a gradual voltage across the lens diameter. These kinds of lenses have potentially the same applications as conventional fixed lenses, but also have some interesting properties for their practical use, for example, small size, lightweight, low driving voltages, low power consumption and transmissive/reflective operation modes.

**Figure 6:**
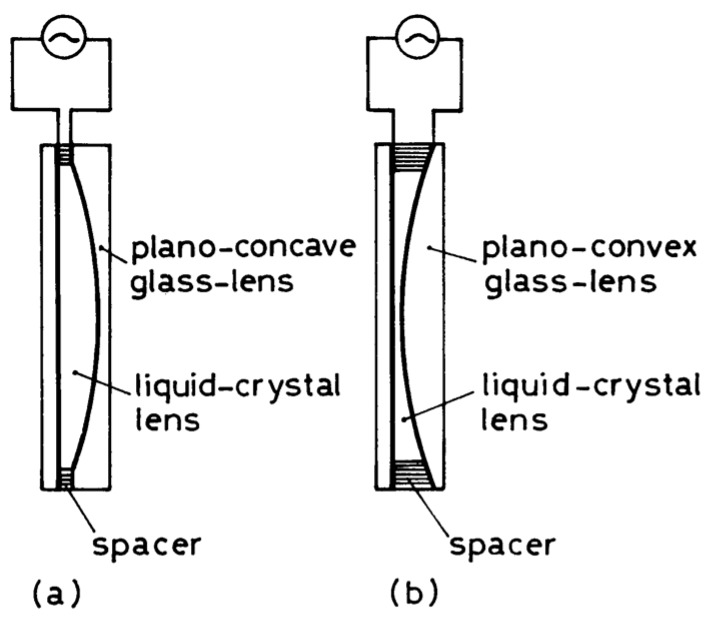
First proposals of LC lenses: (**a**) Plano-concave lens and (**b**) Plano-convex lens [34]. The Japan Society of Applied Physics (JSAP). Reprinted with permission from Sato, S. Japanese Journal of Applied Physics; published by IOP Publishing, 1979.

At the end of the 1970s, some pioneer research, which gave rise to the first proposals of adaptive lenses based on LC materials, were carried out. Berreman *et al.* with patent application in 1977 [33] and Sato *et al.* in 1979 [34] (Figure 6) were the pioneers. The first adaptive lens was formed by a cavity of glass with a certain curvature filled with LC. This device had a low response time due to the LC layer thickness. In addition, another problem was the molecular orientation within this thick and curved cavity. This is the reason why this particular technique was not further developed. 

Two years later, in 1981 [35], a group from Syracuse University made a LC cylindrical lens with electronic control working as an adaptive optical element. This type of lens employed a large number of electrodes to achieve a proper voltage gradient. This new concept of lens gave rise to other research works [36] and subsequently to spherical lenses [37].

At the end of the 1980s, new research works demonstrated LC lenses with micrometric diameters for the first time. First references are from Sato *et al.* [38,39]. In this time, the first Fresnel lenses were also proposed [40]. This configuration reduced the required thickness and increased the achievable diameters.

Even though LC lenses were first reported more than 30 years ago, they remain an active field of research today. In general, the applications of LC lenses, regardless of whether they are microlenses or not, are numerous, as are the applications of fixed lenses or GRIN lenses, with the great advantages in weight and volume reductions and a focal length tunable by voltage. For instance, LC lenses have been proposed to work in imaging systems of portable devices. In this field, LC lenses can help realize auto-focusing systems and optical zoom systems for portable devices, such as cell phones and cameras [41]. In addition, it has been proposed for pico-projection systems, helping to electrically adjust the focusing properties of the projected image without a mechanically adjusting position of a projection lens [42]. In the case of holographic projection systems, a LC lens can also help to correct the mismatch of the chromatic image size which is very important for a full-color holographic projection system [43]. Liquid crystal lenses can also be used as concentrators and a sun trackers in concentrating photovoltaic (CPV) systems [44].

It is worth mentioning the field of bio-optics, for example, the use of lenses for medical instrumental in applications like endoscopy. A LC lens can be adopted to electrically enlarge the depth-of-field of the endoscopic system [45]. Moreover, the lens power of LC lenses is not only electrically tunable, but it can also be positive or negative, which has interest for ophthalmic lenses. In this case, lenses with millimeter diameter are required [46]. Liquid crystal lenses can correct myopia impact. Commercial glasses based on LC lenses were developed in 2011. They have a switchable focal distance [47] solving the problem for patients that require different types of glasses for different activities. Unfortunately, the company went bankrupt this year (2015) due to a high rate of return (battery issues and some defective devices). Liquid crystal lenses can also be a kind of “extra-artificial crystalline lens” to compensate the degradation of the crystalline lens of aging eyes or eye accommodation [48]. Some important advances in this field can be produced by LC lenses working with unpolarized light [49], which produces a considerable increase in the optical efficiency.

In short, in recent years, there have been lots of developments tailored to the needs of different applications. Over this period, many new topologies have been proposed, such as polymer gel stabilization, patterned electrode, curved surface, Fresnel lens or modal control. At micrometric scale, and for use in autostereoscopic applications, the following should be highlighted: curved surface, patterned electrode and modal control. The next three sections study these topologies in detail. Finally, the last section summarizes the different fabrication methods and characteristics of these topologies.

### 3.1. Curved Surface

**Figure 7:**
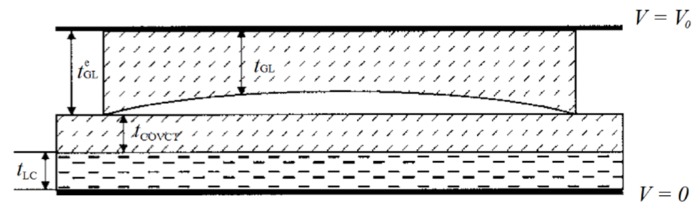
Multilayer liquid crystal lens [53]. Reprinted with permission from Wang, B.; Ye, M.; Sato, S. Applied Optics; published by OSA Publishing, 2004.

**Figure 8:**
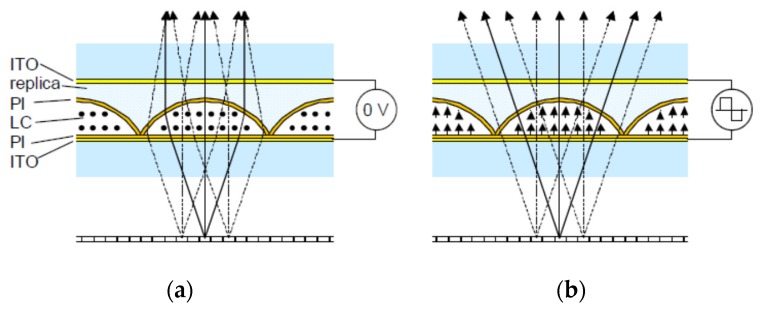
Active birefringent lens: (**a**) without voltage and (**b**) with voltage [55]. Reprinted with permission from Willemsen, O.H.; de Zwart, S.T.; IJzerman, W.L.; Hiddink, M.G.H.; Dekker, T. International Society for Optics and Photonics; published by SPIE, 2006.

This category includes all lenses having at least a non-planar surface. Most of the autostereoscopic prototypes based on LC microlenses use this topology. The criteria of classification of lenses from the following list is based on the type of structure and material used in the fabrication process:

- *Curved ITO*: Consists of a curved electrode of indium titanium oxide (ITO). This structure has the same problems as dual-voltage lenses which require a complex fabrication process [50]. Moreover, no reports at micrometric scale have been reported. For these reasons, they are less relevant to this list.
- *Curved glass*: These lenses were the first LC lenses, a cavity of glass [51]. Although it could be guessed that this old design would be overpassed by new technologies, it is, surprisingly, still used. The main reason is the application of this design to autostereoscopic devices. As mentioned above, this particular technique was not further developed because it had low response time and problems of lack of homogeneity. However, new proposals have reported LC to work as microlenses (immersed polymer microlenses) [52]. This reduces the necessary thickness. Moreover, techniques of LC multilayers further reduce the thickness and the inhomogeneity (Figure 7). When a dielectric layer and a LC layer are sandwiched between two continuous electrodes, the thickness variance of the dielectric layer gives rise to an inhomogeneous electric field in the LC layer [53].

- *Curved Photoresin*: In this approach, the photoresin has a spherical or cylindrical shape and is surrounded by the LC material [54]. Its main disadvantage is the complex fabrication process. Another option is to produce spherical cavities of photoresin and filled them with LC. This topology has been proposed to work in autostereoscopic devices with switchable 2D/3D mode:
  ➢ *Active birefringent lenses*: A typical structure is shown in Figure 8 [55]. The cavity formed by the photoresin is filled with LC. Unswitched, a polarized light is affected by the extraordinary refractive index, greater than that of the photoresin (a positive lens is formed). When the LC is switched by an external electrical field, the polarized light is affected by the ordinary refractive index, similar to that of the photoresin (the light passes through without deviation). The main disadvantage is the high operating voltage (50–100 V).

**Figure 9:**
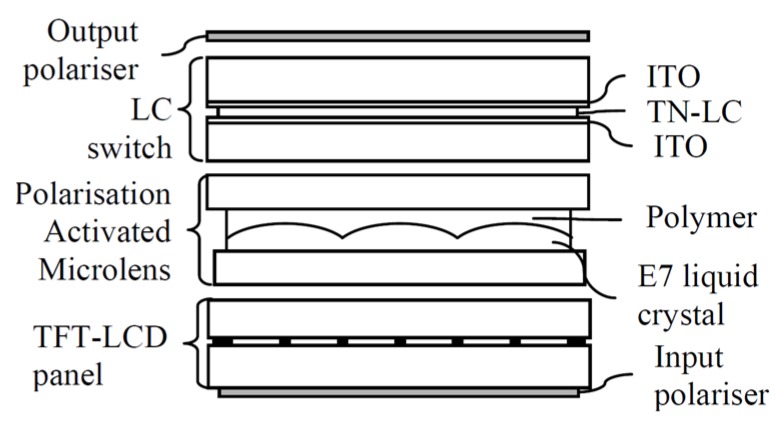
Polarization activated microlenses [22]. Reprinted with permission from De Boer, D.K.G.; Hiddink, M.G.H.; Sluijter, M.; Willemsen, O.H.; de Zwart, S.T. International Society for Optics and Photonics; published by SPIE, 2007.

  ➢ *Polarization Activated Microlenses*: In this structure, two LC layers are used (Figure 9) [22]. One layer acts as the lens (nematic LC) and the other control the polarization by a twisted nematic (TN) LC. The TN LC cell change the polarization of the incoming light so it is affected by the extraordinary or ordinary refractive index of the nematic LC layer. This reduces considerably the operating voltage (only the TN cell is switched) but complicates the fabrication process. This type of lens only switches between focusing and non-focusing states, rather than tuning in a continuous focusing range like other LC lenses. For this reason, this structure has been proposed to work in a switchable 2D/3D mobile phone display [56]. Another option is to create polymeric lenses and use a similar structure [57]. The advantages are the switching speed and a reduction in crosstalk.

In summary, curved surface topology has the problems of high operating voltage or complex fabrication process. Liquid crystal alignment becomes a challenge near the curved surface. Conventional techniques like mechanical rubbing and photo-alignment do not provide satisfactory alignment quality on the curved surface. To overcome these problems, patterned electrode technique could be used. It makes use of the fringe fields produced through a dielectric layer to produce a phase profile within a LC lens. The optical properties depend on the relation between the lens aperture and the LC thickness. This configuration requires lenses with diameters smaller than 200 µm (it depends on the LC layer thickness). This technology is explained in the next section.

### 3.2. Patterned Electrode

**Figure 10:**
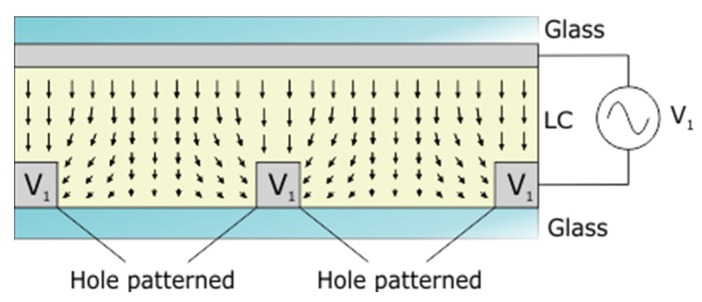
Structure of a liquid crystal microlens based on hole patterned technique.

As mentioned above, patterned electrode technique was the first proposed method to obtain LC lenses. Despite this, this technique has reinvented itself and is still present in many novel proposals. At the beginning, only a series of electrodes in contact with the LC were used to create a voltage gradient [58]. The first microlenses were fabricated by Nose and Sato in 1989 using hole patterned technique [38]. This technique is made up by several patterned circular holes in the upper electrode (Figure 10). If the diameter is small enough, the fringe fields are capable of generating a lens-like phase profile in the LC layer. Years later, other models were proposed based on the inclusion of a dielectric between the electrode and the LC layer. Thanks to this configuration, the fringing field near the electrode edges (that causes unwanted LC twist alignment) is avoided. This technique, proposed in 2004, was known as dual voltage [59]. The main disadvantage was the high operating voltage (>50 V) caused by the space between the electrode and the LC. When a high optical power is required, multilayered structures could be used [48].

Recently, a lot of research based on this technology has been presented. Most of them place the electrodes next to the LC layer as the first microlens (Figure 10). For example, tetragonally hole-patterned electrodes [60]. In that work, each LC cell in the lens array behaves like a cylindrical or spherical lens by electrically adjusting the applied voltages. Another recent proposal is a LC lens with two divided and double circularly hole-patterned electrodes [61]. The radially and axial symmetrically distributed refractive index of the hole-patterned aperture can be obtained by controlling the two divided circularly hole-patterned electrodes and the double circularly hole-patterned electrodes. 

The requirement of working with polarized light is an issue that reduces the final brightness in autostereoscopic displays based on LC microlenses. Polarization independent adaptive microlenses have also been proposed. These proposals avoid the use of polarizers increasing the optical efficiency. There are different approaches, including blue-phase LC [62] and nematic LC [63]. Circular hole-patterned electrodes with tunable coaxial bifocals (CB) synthesized via photopolymerization of LC cells have also been demonstrated recently [64]. Despite the photo-polymerization method, the bi-axial confocal LC lens can also be fabricated by hole-patterned structure [65]. Devices manufactured with this method consist of two types of tunable CB LC lenses fabricated via different photocurable processes. Another recent proposal has been a hole patterned electrode structure with ultrathin glass slabs [66].

In the field of autostereoscopic devices, some interesting proposals have been made recently. For example, a scanning Multi-electrode Driven Liquid Crystal (MeD-LC) lens, achieved to reproduce several views in a 3D display [67]. This configuration is based on a parallel electrode pattern. By applying a sequentially voltage, a lens-like shape is formed under the electrodes with voltage. Thus, the lens could move (shift) sequentially on the horizontal direction to project the images to different viewing angle. Another work propose the use of patterned microlenses with sizes similar to the pixel pitch to obtain a full resolution autostereoscopic display [68]. Another original contribution is the proposal of this type of lenses to work in autostereoscopic devices, demonstrating the focal length tunability, the deviation of images [69] and the tuning of the 3D distance. Some recent devices have the ability to display vertical and horizontal views; to achieve this function, a hole patterned electrode on each substrate has been manufactured. The substrates have been arranged with the comb type electrodes in orthogonal position, besides complex electrical signals are used [70]. The result is a cylindrical LC microlens array with rotary optical power and tunable focal length capability. A focal distance tunability ranging from 0.2 to 1.2 mm in a 125 µm lens is obtained. This would produce a 3D distance from 20 to 80 cm, perfect for a mobile application. It is important to note that this new configuration also leads to minimum aberrations. In the case that some aberrations appear, a tunable compensator device based on hole patterned electrode has been demonstrated in [29]. It is demonstrated how some classical aberrations as spherical or coma are controlled with voltage more than –1 and 2 waves, respectively. Finally, other research works have been focused on modeling the electric field distribution in order to have optimal designs [32,71]. These studies reveal a critical interrelation between the structural parameters of the microlens in order to distribute the voltage with a parabolic profile. This study concludes that depending on the LC birefringence, a ratio of 2.4 between thickness and diameter of a microlens is stablished for MDA 98-1602 nematic LC from Merck is necessary to manufacture microlenses. For example, in case of multiview displays for diameters of 100 µm, the necessary thickness would be 42 µm. Large thicknesses could limit the switching speed of the final device.

Due to the constant reduction of the pixel size, it seems evident that this topology is going to be protagonist in future products. Despite this, as mentioned above, there are certain cases where the necessary thickness is too thick. One technique that could be used to correctly distribute the voltage in this specific case is modal control.

### 3.3. Modal Control

**Figure 11:**
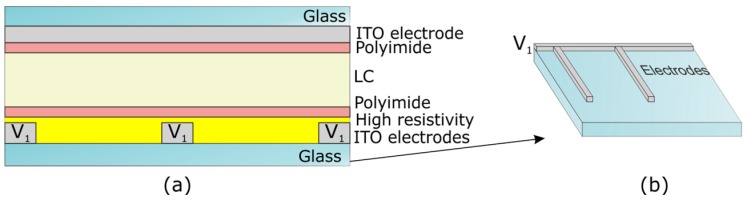
Structure of a liquid crystal cylindrical lens based on modal control technique: (**a**) 2D view and (**b**) 3D view of the bottom substrate, detail of the electrode pattern.

Modal control technique has become an alternative for lens design because overpasses the main drawbacks of previous techniques. A key advantage is the driving method, with only one voltage control at very low values (<3 V). Moreover, when the established ratio between the microlens diameter and thickness is not fulfilled, using this technique instead of patterned electrode configuration is recommended (the fringe fields do not reach the microlens center). First research on modal control was reported by Naumov *et al.* [72]. This technique consists of generating a radial graded refractive index across the lens aperture by using a layer of high sheet resistance (MΩ/sq), deposited onto the pattern electrode, as a control electrode. Sheet resistance of the control electrode is a key design parameter. Its value must be in the range 100 kΩ/sq to few MΩ/sq for lens diameters on the order of millimeters [73]. This layer creates a voltage divider with the LC impedance causing a hyperbolic voltage gradient across the lens aperture [17]. Different materials have been reported to act as a control electrode layer, Poli(3,4-etilenodioxitiofeno) (PEDOT) [74], thin ITO layers [75] (10 MΩ/sq [76]) and titanium oxide films [77] are some of them. A diagram of a modal liquid crystal cylindrical lens is depicted in Figure 11.

Indeed, it is not a simple task to obtain exact thicknesses of the high impedance layer; setting a thickness value must offer a good compromise between impedance (which affects lens refractive index gradient) and transparency (which influences lens quality). On the other hand, control voltages must have optimized the amplitude, frequency and shape. Due to the capacitive effect of the LC, LC layer impedance is frequency dependent. In addition, one of the main drawbacks of systems using these optical arrangements is the generation of aberrations. Thus, the suitable choice of the voltage shape (set of harmonics) can minimize phase aberrations caused by both, the non-ideal distribution of the electric field and the nonlinear electro-optic response of the LC with voltage [77]. For this reason, some systems that control the frequency, amplitude and duty cycle have been proposed to control the phase profile [78]. Novel structures, based on symmetric electrodes, have also been proposed to reduce aberrations [74]. In this case, spherical aberration range from −0.4 to 0.4 waves and comma range from −0.2 to 0 waves. One application worth mentioning is a wavefront modulator based on modal control technique [79]. In addition, a modal LC lens is commercially available at Flexible Optical B.V. [80].

Unfortunately, the fabrication of modal lenses at micrometric scale implies a technological challenge. The main problems are difficulties in finding layers of 0.1–1 GΩ/sq and the fact that, at some point, the fringe fields distributed by LC are sufficient to create a voltage distribution without the need of a high-resistivity layer. 

For autostereoscopic applications, there have been several proposals. In [81,82], a system that enables rotation of the 3D image is demonstrated using this approach (Hi-R LC). Despite this, the resistivity is not high enough to create a voltage gradient when all electrodes are at the same voltage. They need a ground voltage at the center of each lens to have a voltage gradient, reducing the advantage of tunability of modal LC lenses. Other works, propose the use of polymer stabilized blue phase LC materials to obtain fast-response LC lens for 3D displays [83]. Another advantage of these types of systems is that they are polarization independent.

Recently, a cylindrical microlens array with a new manufacturing protocol based on a set of two layers (a dielectric layer of SiO_2_ and a metallic layer of nickel) for lenses with modal driving was reported [27]. This array consists of a set of lenses, each 570 μm in diameter whose behavior is based on a nanometric layer of nickel. This layer determines the voltage distribution across the lens diameter; without it, each lens would work as a hole-patterned lens and the voltage would drop at the lens edges. Modal lenses exhibit sometimes some spherical aberration because of the high resistivity of the manufactured layer. However, this aberration can be reduced by increasing the thickness of the metallic layer [84]. The lenses of this contribution were demonstrated to work in an autostereoscopic system and validated through 3D contrast measurements. A 3D distance of 30 cm is obtained for 7.5V_RMS_. Unlike other proposed systems [22,51], modal LC lenses have a wide range of focal length tunability, so these systems could adjust the 3D distance over a wide range.

As mentioned above, the necessary sheet resistance is too high in modal microlenses. In order to achieve this value using Nickel, the percolation limit has to be reached. Another option is the use of amorphous Silicon (instead of SiO_2_). As it is reported in [85], when a layer of amorphous Silicon is sputtered with Nickel and then is crystallized (with high temperature), the sheet resistance reaches GΩ/sq [86].

### 3.4. Fabrication Methods of Liquid Crystal Microlenses for Autostereoscopic Applications

In this section, a comparative study of various methods of microlenses fabrication and some important characteristics, described in Section 3.1, Section 3.2 and Section 3.3, is included in Table 3. This table describes the type, topology, certain keys of the fabrication process, the necessary voltage to have the optical power, the switching speed and the requirement of a polarized light source.

**Table 3:** Comparison between different LC microlenses characteristics.

Type	Topology	Fabrication Process	Voltage 3D	Switching Speed	Polarized Light
Curved Surface	Curved ITO	Sputtered ITO	40–140 V_RMS_	~1 s	Yes
	Curved Glass	Immersed polymer microlenses	10–20 V_RMS_	~2 s	Yes
	Curved Photoresin Active	Complex fabrication process, several layers and TN LC in active cells	Depend on the topology	Several seconds	Yes
	Curved Photoresin Passive		3.3 V_RMS_	<100 ms	Yes
Patterned Electrode	Hole patterned	Simple holes patterned	2–3 V_RMS_	Several seconds	Yes
	Bi-axial confocal	Special alignment layers	3–10 V_RMS_	Unknown	No
	Blue-Phase	The blue-phase has to be synthetized	50–100 V_RMS_	Several µs	No
	MeD-LC	Multiple electrodes	5–10 V_RMS_	<100 ms	Yes
	Rotary cylindrical	Orthogonal electrodes	2–3 V_RMS_	Several seconds	Yes
Modal control	Hi-R LC	Difficult to obtain the high resistivity layer	~2 V_RMS_	~0.6 s	Yes
	Cylindrical microlenses		~7.5 V_RMS_	Several seconds	Yes

## 4. Concluding Remarks and the Future of Autostereoscopic Displays

Thanks to the commercial success of some stereoscopic film productions, 3D vision has acquired a growing importance in audiovisual industry in the past ten years. This great commercial impact has produced an exponential increase in 3D vision research. The technological transfer to commercial applications is bigger than in any other field. Despite this, the first generation of autostereoscopic displays failed to generate enough consumer excitement. The main reasons were little 3D content and performance issues. In this sense, the use of LC microlenses brings some important improvements. There are numerous techniques for manufacture LC lenses that could be of clear advantage to use in autostereoscopic displays. For this reason, some topologies of LC microlenses that were in the research stage only a few years ago, will be on the market for large displays soon. For example, curved surfaces seem to be used in a future release of a 15″ autostereoscopic display. Patterned electrode is the most promising for small pixel sizes, while modal control could be used for multiview displays where the required microlenses have diameters bigger than the previous technique.

In the last year, the use of portable devices has grown rapidly. Everything indicates that in the near future most of the multimedia content will be displayed in mobile phones or tablets. For this reason, many experimental and theoretical research groups worldwide have actively worked on alternative solutions for 3D vision in portable devices in which no external devices are required. For these kinds of devices, some aspects have to be taken into account:

- The observer usually has the device in his own hands, so the 3D distance is considerably lower than with big displays. This requires a high optical power.
- Every observer has different physical characteristics, so tunability of this distance is especially required for a useful device.
- Aberrations of the optical elements used in this type of systems are a problem that has to be solved in order to reduce the crosstalk.
- The ability to display vertical, as well as horizontal, views is an added value of autostereoscopic displays.

Liquid crystal lenses are excellent candidates to implement alternative solutions: first, for mitigating some of the identified drawbacks; and second, for creating new gadgets with new capabilities that are not trading currently in the market. The high optical power requirement can be solved using thick samples or multilayered lenses when the response time is critical. The tunability is an inherent characteristic of some configurations, such as patterned electrode. Aberrations can be compensated for with some novel devices also based on LC. The ability to display vertical and horizontal views, while maintaining the tunability characteristic, has been recently solved. In conclusion, the use of spatial multiplexing technique would be possible in portable devices when LC devices are used.

Despite this, the future of autostereoscopic displays seems to be with full parallax and multiview displays. One technology that displays 3D light is integral imaging. Liquid crystal lenses could be of important use in integral imaging. The use of spherical arrays of patterned electrode microlenses with different focal lengths, tunable and controlled aberrations could be the key to achieve displays with high enough quality to reach the market. Still, spatial multiplexing has the bottleneck of final resolution, something that probably could be solved in the future with the introduction of pixels with nanometric size.
